# Compact All-Fiber SERS Probe Sensor Based on the MMF-NCF Structure with Self-Assembled Gold Nanoparticles

**DOI:** 10.3390/s25175221

**Published:** 2025-08-22

**Authors:** Peng Cai, Tiantian Xu, Hangan Wei, Huili He, Fu Li

**Affiliations:** 1 Key Laboratory of Ecosystem Carbon Source and Sink, China Meteorological Administration (ECSS-CMA), Wuxi University, Wuxi 214105, Chinatt.x@nuist.edu.cn (T.X.); hehuili0902@163.com (H.H.); 2 Sino-Belgian Joint Laboratory of Geo-Information, 9000 Gent, Belgium; 3 Key Laboratory of Embedded System and Service Computing, Tongji University, Ministry of Education, Shanghai 201804, China; 4 Jiangsu Key Laboratory of Atmospheric Environment Monitoring and Pollution Control (AEMPC), Collaborative Innovation Center of Atmospheric Environment and Equipment Technology (AEET), Joint International Research Laboratory of Climate and Environment Change (ILCEC), School of Environmental Science and Engineering, Nanjing University of Information Science & Technology (NUIST), Nanjing 210044, China; ha.w@nuist.edu.cn

**Keywords:** surface-enhanced Raman scattering, brain natriuretic peptide, heart failure, microfluidic chip, fiber-optic sensor

## Abstract

Brain natriuretic peptide (BNP) is an important biomarker for the diagnosis and prediction of chronic heart failure (CHF). Aiming at the problems of the low sensitivity and poor portability of traditional BNP detection methods, this study proposes a Surface-enhanced Raman-scattering (SERS) fiber-optic sensor based on a multimode fiber (MMF)–no core fiber (NCF) structure. The sensor achieves BNP detection by significantly amplifying the Raman signal of the toluidine blue (TB) marker through the synergistic effect of NCF’s unique optical transmission modes and localized surface plasmon resonance (LSPR). To optimize the sensor performance, we first investigated the effect of the NCF length on the Raman signal, using Rhodamine 6G (R6G), and determined the optimal structural parameters. Combined with the microfluidic chip integration technology, the antibody–BNP–antibody sandwich structure was adopted, and TB was used as the Raman label to realize the quantitative detection of BNP. Experimental results demonstrate that the detection limit of the sensor is lower than the clinical diagnostic threshold and exhibits stability. The sensor sensitivity can be adjusted by regulating the laser power. With its stability and high portability, this sensor provides a new solution for the early diagnosis of heart failure and demonstrates broad application prospects in biomarker detection.

## 1. Introduction

Brain natriuretic peptide (BNP) produced by ventricular cardiomyocytes shows significantly increased serum levels in patients with heart failure. This characteristic establishes BNP as a key biomarker for diagnosing and predicting chronic heart failure (CHF) [1,2]. In recent years, with the growing demand for early diagnosis of cardiovascular disorders, BNP detection technology has been evolving towards high sensitivity, portability, and real-time monitoring [3,4]. Currently, the conventional methods for detecting BNP are the enzyme-linked immunosorbent assay (ELISA) and electrochemiluminescence (ECL). These methods generally suffer from complex operation, large equipment size, and poor portability [5,6], making it difficult to meet clinical needs. Promoting point-of-care testing of BNP, improving detection limits, and enhancing system portability have become new trends and key challenges in the development of detection technologies for this critical biomarker of cardiac collapse [7].

Surface-enhanced Raman scattering (SERS) is an effective analytical method that has been extensively applied in sample composition analysis and multi-parameter characterization across various domains, including biomarker identification. This technique demonstrates advantages owing to its label-free approach, operational simplicity, and enhanced detection sensitivity [8]. Nevertheless, the practical implementation of conventional Raman spectrometers employing microscope-based detection systems remains constrained in field applications, owing to their large physical dimensions [9].

Optical fibers are lightweight, small, and low cost. When integrated with SERS technology, these features enable enhanced system flexibility, operational reliability, portable deployment, and long-distance detection capacities [10]. The main structures used in fiber-optic SERS sensors are photonic crystal fibers (PCFs), fiber tips, and tapered fibers [11,12]. However, these approaches exhibit significant limitations. Although tapered fibers enhance the evanescent field, their nanoscale tapered regions are mechanically fragile and prone to fracture under stress. Furthermore, fabrication requires precise control over temperature and pulling speed, resulting in complex processes and low yield rates. PCFs, meanwhile, are constrained by intricate microstructures and prohibitively high manufacturing costs [13,14].

To address the above bottlenecks, we propose an optical fiber SERS sensor based on a multimode fiber (MMF)–no core fiber (NCF) structure for BNP detection. The sensor integrates MMF and NCF through fusion splicing to form a probe [15]. This structure is more stable and easier to fabricate than taper fibers. Additionally, a self-assembly process is used to securely construct the SERS active substrate on the NCF surface [16], avoiding the use of hazardous chemicals such as piranha solution. The coreless architecture of the NCF leads to a unique optical field distribution during light transmission. The optical field energy tends to concentrate towards the fiber surface, significantly enhancing the near-field intensity at its surface [17,18]. The all-fiber integrated design improves portability and enables remote detection [19]. This approach effectively addresses the environmental adaptability, integration, and operational stability issues found in photonic crystal fibers and tapered fibers.

In this study, we demonstrate the use of this MMF-NCF fiber-optic SERS sensor for BNP detection. We first prepared gold solution and attached poly(dimethyldiallylammonium chloride) (PDDA) functionalization, AuNPs, and antibody AB1 to NCF sequentially to prepare AFMOF-AuNPs-TB composites; modified them with AB2 antibody; integrated them with a Polydimethylsiloxane (PDMS) microfluidic chip fabricated by a specific process [20,21]; and constructed a detection device consisting of a laser source, a beam-splitting module, and a spectrometer. The BNP molecules are specifically captured by the AB1 antibody on the surface of the AuNPs-MMF-NCF probe. The AFMOF-AuNPs-TB-AB2 complex binds to another epitope of BNP via AB2, forming an antibody–BNP–antibody structure. The unique optical field distribution of the NCF generates an enhanced electromagnetic field near the BNP binding sites. Specifically, the coreless structure of the NCF forms a distinctive optical field distribution during light propagation, effectively intensifying the localized electromagnetic field around the BNP binding sites. Meanwhile, the uniformly distributed gold nanoparticles (AuNPs) on the NCF surface exhibit localized surface plasmon resonance (LSPR) under incident light excitation [22]. LSPR refers to the collective oscillation of free electrons on the surface of metal nanostructures when irradiated by light of specific wavelengths [23]. When the frequency of incident light matches the LSPR frequency of AuNPs, highly localized electromagnetic fields (termed “hotspots”) with intensities much higher than the incident light field are generated on and near the nanoparticle surfaces. Raman label molecules located at or near these “hotspots” exhibit significantly enhanced excitation efficiency and radiation efficiency of scattered Raman photons due to the LSPR effect, resulting in intense surface-enhanced Raman-scattering signals. In this study, quantitative analysis of the BNP concentration is achieved by detecting the characteristic SERS signal intensity of TB. This technique not only improves sensitivity but also enhances portability and practicality, providing new possibilities for clinical applications.

## 2. Materials and Methods

### 2.1. Fabrication Procedure

The preparation process began with the preparation of gold sols, following the method of Frens et al. [24]. Specifically, 1 mL of 1% HAuCl_4_ was added to 100 mL of ultrapure water, which was then heated to boiling with continuous stirring. Once boiling, 1 mL of 1% Na_3_Ct was quickly added and kept boiling with vigorous stirring for 15 min. This process reduces Au^3+^ ions to form AuNPs.

The probe was formed by fusing MMF and NCF, followed by immersing NCF in 0.1% PDDA solution for 30 min. The PDDA molecules were adsorbed onto the surface of NCF through electrostatic interaction, rendering its surface positively charged [25]. The surface of AuNPs prepared by the Frens method was negatively charged due to the carboxylate group generated by the reduction of sodium citrate. The negatively charged AuNPs were bound to the positively charged surface of NCF via electrostatic attraction, forming a monolayer dense arrangement. If the modification time of NCF in the PDDA solution was too short, it would result in insufficient charge density; if too long, it would lead to particle aggregation. When the modification time was 30 min, AuNPs could be uniformly adsorbed on the surface of NCF. If the concentration of AuNPs was too low, it would result in low coverage; if too high, it would lead to particle clustering, reducing the number of “hotspots”. Therefore, it is necessary to maintain the concentration of AuNPs at 0.1–0.5 nM.

**Figure 1:**
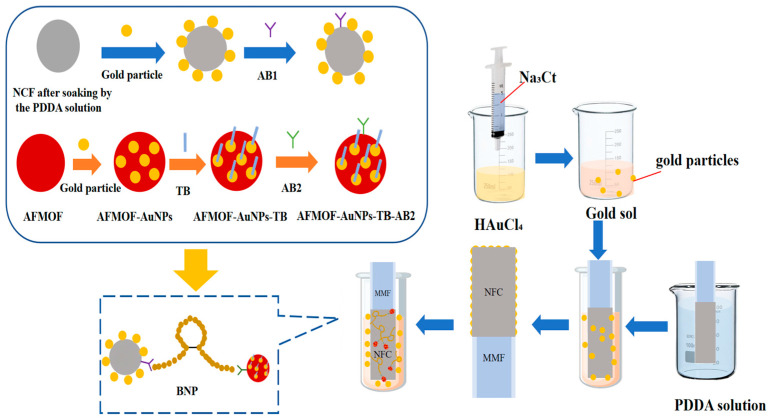
Fabrication and functionalization of the MMF-NCF SERS sensor for ultrasensitive BNP detection.

Amino-functionalized metal–organic frameworks (AFMOFs) served as nanoparticle carriers. AFMOF was incubated with AuNPs for 2 h, achieving surface deposition. The AFMOF-AuNPs composites were immersed in a 1 mM TB solution for 2 h. TB adsorption on AuNPs produced AFMOF-AuNPs-TB complexes. Antibody conjugation was performed on AFMOF-AuNPs-TB complexes. AB2 antibody was covalently immobilized using EDC and NHS chemistry. Meanwhile, the AuNPs-modified MMF-NCF probe was immersed in a solution containing anti-BNP antibody (AB1) for 2 h. AB1 adsorption occurred via combined hydrophobic/electrostatic interactions. The BNP molecules were specifically captured by the AB1 antibody on the AuNPs-MMF-NCF probe, while the AFMOF-AuNPs-TB-AB2 composite binds to another BNP epitope via AB2 to form an antibody–BNP–antibody structure. The entire preparation process is summarized in the flowchart in Figure 1.

### 2.2. Microfluidic Chip Integration

Combining a fiber-optic SERS sensor with a microfluidic chip improves both the performance and practical application [26]. These chips are typically made of plastic-based materials because they are easy to shape, cost-effective, and chemically stable. Polydimethylsiloxane (PDMS) is widely used for fabricating these chips [27]. This silicone material works well with biological samples, is affordable, and can be produced quickly [28].

**Figure 2:**
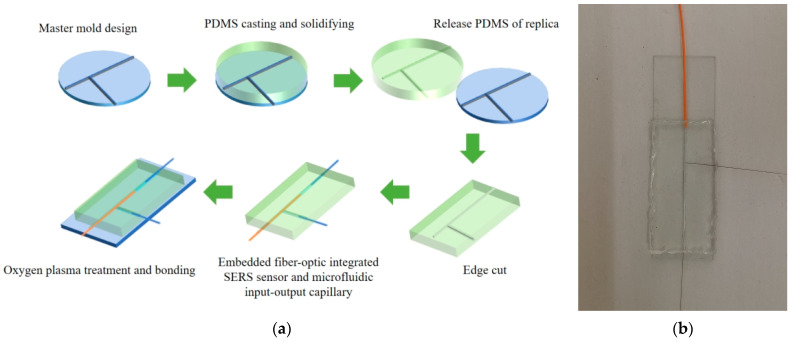
(**a**) Microfluidic chip fabrication process diagram for embedded fiber SERS sensor. (**b**) Schematic diagram of microfluidic chip.

In this study, PDMS was used to fabricate a microfluidic chip. The fabrication process began with the creation of a master mold, based on a pre-designed structure. To prepare PDMS, prepolymer A and crosslinker B were mixed in a 10:1 ratio. The mixture was degassed under low pressure to remove air bubbles and then poured into a mold. The mold was placed in a vacuum oven at 65 °C for 60 min to completely cure PDMS. After curing, the PDMS was removed from the mold and trimmed to obtain the desired microfluidic structure. The PDMS assembly was then plasma treated and bonded to the SERS substrate. The components were then placed in a vacuum oven at 65 °C for 3 h to ensure strong bonding. This process resulted in a functional microfluidic chip with an integrated fiber-optic SERS sensor that was ready for use in the experiments. Oxygen plasma treatment was applied to both the PDMS overlay with microfluidic channels and quartz slides. A fiber-optic SERS sensor, along with the input and output capillaries, was embedded in the microfluidic channel. The PDMS overlay was then securely bonded to a quartz slide. The entire assembly was placed in a vacuum oven for 180 min to further strengthen the bond. Finally, a special UV-activated glue was applied to seal the joints between the optical sensors and fluid channels. All the components of the microfluidic chip, except the input and output capillaries, were sealed with a UV-cured adhesive. The final microfluidic chip is shown in Figure 2a,b.

### 2.3. Experimental Setup

**Figure 3:**
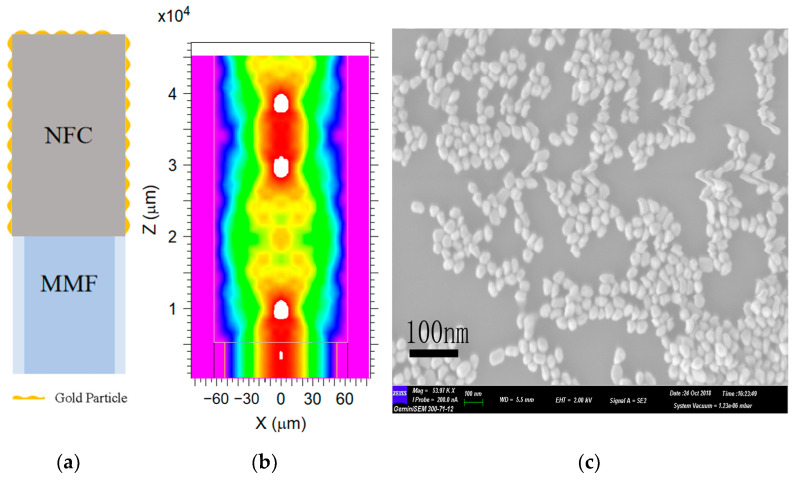
(**a**) Schematic diagram of the sensor. (**b**) Simulation result of light field energy distribution. (**c**) SEM photo of the assembled gold nanoparticles.

A schematic of the fiber-optic SERS sensor based on the MMF-NCF structure is shown in Figure 3a. The core diameter of the MMF was 105 μm, and the cladding diameters of both the MMF and NCF were 125 μm. The energy distribution of the optical field of the MMF-NCF structure simulated using the beam propagation method (BPM) is shown in Figure 3b. The simulation results show that the excitation light can simultaneously propagate along the lateral and end faces of the NCF and interact with the sample near the fiber surface. In addition, a special light transmission mode with significant changes in the beam profile was observed when the NCF length was approximately 5 mm, 25 mm, and 34 mm. This result indicates that a small amount of excitation light was transmitted to the sample region on the outer surface, resulting in a weakened Raman-scattering signal. A scanning electron microscopy (SEM) image of electrostatically self-assembled AuNPs on the NCF surface is shown in Figure 3c. The AuNPs were uniformly distributed (scale bar: 100 nm), with an average particle size of 35 nm and particle spacing of less than 10 nm, which optimized plasmon resonance coupling. This ordered arrangement creates a homogeneous electromagnetic ‘hotspot’ in the sensing region, which is essential for reproducible SERS signal enhancement. By virtue of the unique light transmission mode of NCF (Figure 3b) and the localized surface plasmon resonance (LSPR) effect of uniformly distributed AuNPs on the NCF surface, the problem of uneven nanoparticle deposition on traditional optical fiber substrates is overcome, enabling efficient optical field confinement and near-field amplification, thus significantly improving sensitivity.

**Figure 4:**
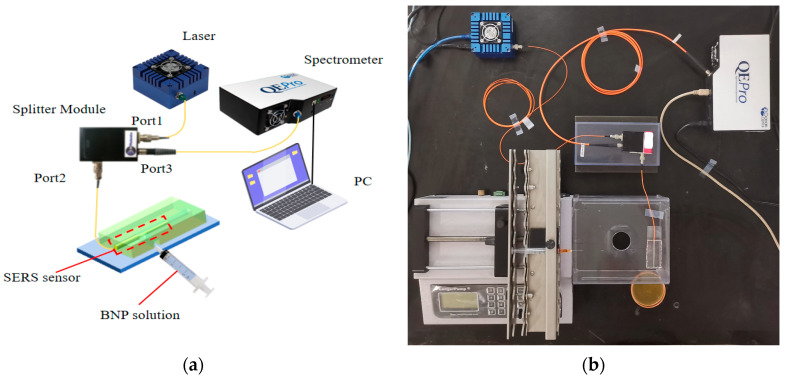
(**a**) Schematic illustration of the experimental setup for detecting Raman-scattered light. (**b**) A real photo of an MMF-NCF SERS sensor.

The experimental setup for detection using the fiber-optic SERS sensor is shown in Figure 4a,b. A 785 nm, 25.1 mW laser (Laser785-5HFUO, OceanHood Optoelectronics Technology Co., Ltd., Shanghai, China) was coupled into the SERS bandpass filter to block the Raman background from the excitation light entering through port 1, whereas a long-pass filter ensures that only the Raman-scattered light probe enters through a customized beam-splitting module (InPhotonics, Inc., Norwood, MA, USA). The module employs a probe from port 2 that passes through port 3. The Raman-scattered light generated by the probe was then measured with a spectrometer (QE Pro, Ocean Optics, Orlando, FL, USA), using an integration time of 10 s.

The SERS sensor with the MMF-NCF structure enables Raman signal transmission through an all-fiber link: The 785 nm excitation light enters the MMF via the beam-splitting module and propagates to the fused NCF region. Here, the intensity of the evanescent wave field on the NCF surface is enhanced due to its specific structural properties. This, in synergy with the LSPR “hotspots” formed by the AuNPs modified on the surface, significantly amplifies the scattering signal of the adjacent Raman label molecule TB. The generated Stokes Raman photons are coupled into the NCF through the evanescent wave field, propagate backward along the NCF, pass through the fusion interface into the MMF core, and stably transmit back to the initial end of the fiber. After re-entering the beam-splitting module, the signal is processed by a bandpass filter to remove background noise and a long-pass filter to select Raman-scattering light above 785 nm. Finally, the filtered signal is focused by a lens onto the spectrometer (QE Pro, Ocean Optics, Orlando, FL, USA), where it is dispersed and detected to obtain the characteristic spectrum of TB, enabling highly sensitive quantitative detection of BNP.

To detect BNP, BNP solution was injected into the inlet port of the microfluidic chip. When the sample flowed through the surface of the MMF-NCF probe modified with AuNPs, the BNP molecules were trapped by the AB1 antibody adsorbed on the surface of the probe to form a probe surface-immobilized AB1-BNP complex. A solution of the AFMOF-AuNPs-TB-AB2 complex was injected and delivered to the probe area through the microfluidic channel. The AB2 antibody binds to another epitope of BNP to form a stable AB1-BNP-AB2 structure, which brings the TB molecule in close proximity to the probe surface through AuNPs. A 785 nm laser (Laser785-5HFUO, 25.1 mW) was used to couple the fiber-optic probe via a custom beam-splitting module (InPhotonics). The excitation light is delivered to the NCF region via optical fiber, leveraging the NCF’s distinct optical transmission modes (Figure 3b) to generate an enhanced localized optical field at the BNP binding sites. Simultaneously, the uniformly distributed AuNPs on the NCF surface significantly amplify the Raman signal from the TB molecules through Localized Surface Plasmon Resonance (LSPR) effects. The Raman-scattered light was returned from the same optical fiber to the beam-splitting module, which was detected by the spectrometer after passing through the bandpass and long-pass filters. The characteristic peak intensity of TB molecules is used as the detection index, and the quantification of the BNP concentration is achieved by standard curve.

## 3. Results and Discussion

**Figure 5:**
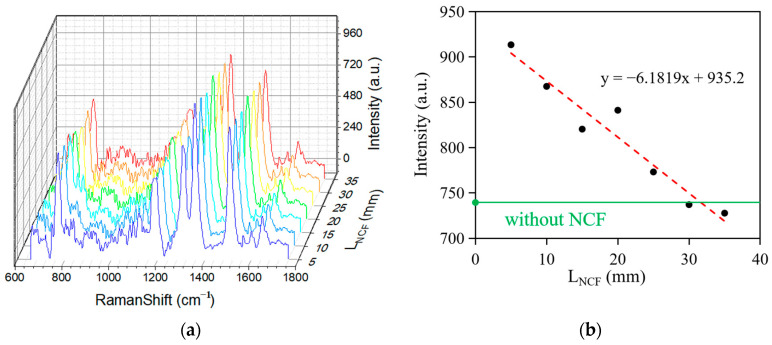
(**a**) The Raman spectra of R6G in water with different lengths of the NCF. (**b**) The relationship between the Raman peak intensity and L_NCF_ (length of the NCF).

In this experiment, we first investigated the relationship between the Raman peak intensity and NCF length. Figure 5a shows the Raman spectra of Rhodamine 6G (R6G) at different NCF lengths after baseline correction using the wavelet transform and iterative fitting method. As shown in Figure 5b, the overall Raman peak intensity tends to decrease with increasing NCF length, which may originate from the accumulated loss during optical transmission. However, the Raman signal intensity of the 20 mm length NCF was significantly higher than that of the adjacent 15 mm and 25 mm data points. This anomalous phenomenon originates in distinctive optical transmission modes within the NCF: when the NCF length reaches the range of 25–35 mm, the optical energy is focused in the central region of the optical fiber, leading to a reduction in the effective interaction region between the excitation light and the sample molecules, thus weakening the enhancement effect of the Raman signal. The existence of this phenomenon is of significant importance for further research on the optimization of Raman-enhanced signals and fiber-length design.

**Figure 6:**
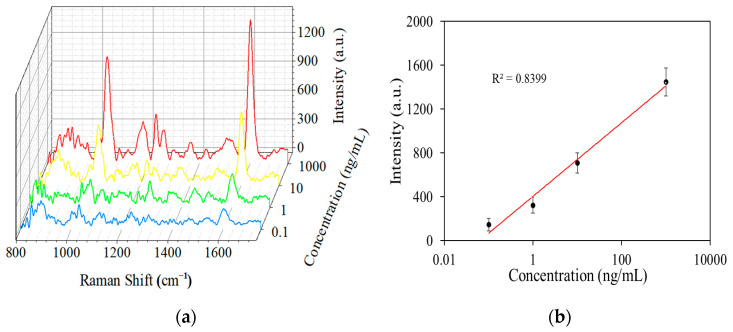
(**a**) Three-dimensional waterfall plot of Raman spectra of BNP solutions at various concentrations. (**b**) Linear relationship between Raman signal intensity and concentration of BNP solutions.

We then measured BNP levels using a fiber-optic SERS sensor based on an MMF-NCF structure, that is, an antibody–BNP–antibody combination consisting of AuNPs-MMF-NCF-AB1, BNP, and AFMOF-AuNPs-TB-AB2. The concentration of BNP is roughly proportional to the Raman signal intensity of the TB molecule, owing to the antibody–antigen interaction. Figure 6a shows the Raman spectra of BNP at different concentration ranges under a 40 mW laser. It can be observed in Figure 6a that the intensity of the Raman peak increases significantly with increasing concentration. The Raman peak can still be detected at a solution concentration of 0.1 ng/mL. Figure 6b presents the linear relationship between the 1592 cm^−1^ peak intensity and the concentration of BNP. As shown in Figure 6b, the Raman peak can also be detected at a solution concentration of 0.1 ng/mL. After multiple repeated experiments, the linear fitting error was found to be <5%, and the goodness of fit (R^2^) was 0.8399, indicating that the MMF-NCF fiber SERS sensor possesses high sensitivity and high reliability, enabling it to meet the requirements for BNP detection and early diagnosis of heart failure.

**Figure 7:**
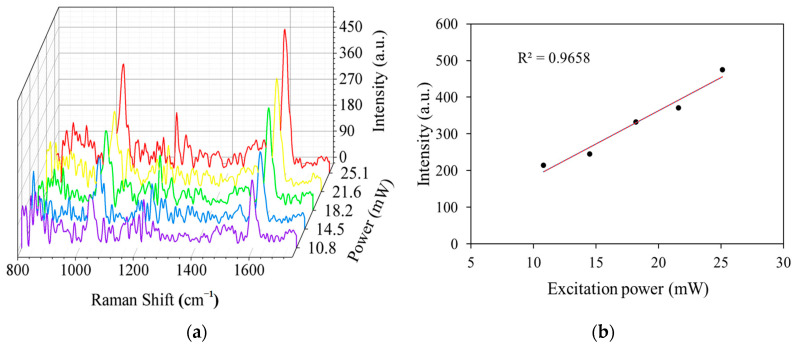
(**a**) Three-dimensional waterfall plot of Raman spectra under different excitation powers. (**b**) Relationship between excitation power and Raman signal intensity.

Figure 7a shows the Raman spectral waterfall plots of the BNP sample solutions with a concentration of 10 ng/mL at different laser powers. The intensity of the Raman signal was significantly enhanced with increasing laser power. Figure 7b shows the relationship between the excitation power and the intensity of the 1592 cm^−1^ trait peak, where the goodness of fit (R^2^) was 0.9658. The results show that the Raman signal intensity is positively correlated with the laser power. This indicates that an appropriate increase in laser power not only enhances the intensity of the signal but also improves the detection of low-concentration samples. Therefore, the regulation of laser power is one of the key factors for optimizing the Raman signal intensity and achieving high-sensitivity detection, especially when very low-concentration samples need to be detected, and power regulation becomes a necessary means to enhance the sensitivity.

## 4. Conclusions

In this study, we present a fiber-optic SERS sensor based on the MMF-NCF structure for highly sensitive detection of BNP. This sensor harnesses the synergy between the localized optical field enhanced by NCF’s distinctive transmission modes and the LSPR effect, significantly achieving indirect detection of BNP by amplifying the Raman signal from the TB labels. The construction of AuNPs on the NCF surface by electrostatic self-assembly further optimized the performance of the SERS substrate, significantly improving the detection of Raman signals. After integrating this sensor with the microfluidic chip, the system demonstrated convenient, compact, and stable features that enhanced the operability and practicality of the device. The sensor has high sensitivity for low-concentration detection, and the signal intensity can be effectively enhanced by adjusting the excitation power. This design provides a new approach for the early diagnosis of chronic heart failure (CHF) and demonstrates the potential of SERS technology for a wide range of biomarker detection applications. In the future, sensors can be optimized to meet the needs of more disease markers, which will promote the practical application of this technology in medical diagnosis.

## Data Availability

The data are available from the corresponding author on reasonable request.
